# Genetic Approach for Joint Transmission Grouping in Next-Generation Cellular Networks

**DOI:** 10.3390/s22197147

**Published:** 2022-09-21

**Authors:** Yu-Po Kuo, Ya-Ju Yu, Tzung-Pei Hong, Wei-Kuang Lai

**Affiliations:** 1 Department of Computer Science and Engineering, National Sun Yat-sen University, Kaohsiung 804, Taiwan; 2 Department of Computer Science and Information Engineering, National University of Kaohsiung, Kaohsiung 811, Taiwan

**Keywords:** CoMP, joint transmission, cache, backhaul, 5G cellular networks

## Abstract

Coordinated multipoint joint transmission (JT) is one of the critical downlink transmission technologies to improve network throughput. However, multiple cells in a JT group should have the same user data to transmit simultaneously, resulting in a considerable backhaul burden. Even when cells are already equipped with caches in fifth-generation networks, JT groups, without effectively utilizing the caching data, still cause unnecessary backhaul data traffic. In this article, we investigate the JT grouping problem with the consideration of caches at cells. Then, we propose a genetic approach to solve the above problem with the objective of minimizing the amount of backhaul data traffic subject to the data-rate requirement of each user. The simulation results show that our proposed generic algorithm can significantly decrease the backhaul bandwidth consumption compared to the two baselines.

## 1. Introduction

With improvements in smartphone screen resolution, the requirement for high video quality has increased. According to Cisco’s data traffic forecast, by 2023, fifth-generation (5G) networks will generate approximately three times more traffic than fourth-generation (4G) networks [1]. Advanced wireless communication technologies need to support vast video traffic to increase network throughput, particularly for cell-edge users.

To achieve a higher network throughput, the third-generation partnership project (3GPP) introduced coordinated multipoint (CoMP) transmission and reception to mitigate inter-cell interference and improve spectrum efficiency [2]. The CoMP is an essential technology for 5G [3,4] and future wireless networks. Two coordination schemes can be used for downlink–joint transmission (JT) and coordinated scheduling/beamforming (CS/CB). In the CoMP-JT, multiple coordinated cells transmit an application file simultaneously to the user equipment (UE). The superimposed signal enhances the signal strength and further increases the data transmission rate. In the CS/CB, each user connects to a single cell [5]. This article focuses on the CoMP-JT because it generally performs better than the CS/CB [6]. However, in the CoMP-JT, several cells must join a JT group to transmit the same file at the same time and frequency, resulting in heavy backhaul traffic. The performance of the CoMP-JT is significantly degraded if the backhaul capacity cannot transmit extremely duplicated data traffic.

Therefore, the diminishing backhaul traffic from the JT has been studied [7,8]. Some works [9,10,11] have considered caching files in cells. If the file requested by a UE is stored in the cells, the cells can use the JT to transmit data to the UE without fetching it from cloud servers so that the backhaul bandwidth is saved [12]. However, if we do not consider the caching status of the cells to select the JT groups, then the cells quickly exhaust the radio resources without suitably using the caching files, increasing the backhaul traffic. Consequently, this article investigates the JT grouping problem considering cells with caching files.

The JT grouping problem is a challenging issue in the CoMP [13]. The JT grouping problem initially considers a limited backhaul bandwidth as a constraint for optimizing different objective functions [14,15,16,17,18]. Some studies have attempted to minimize backhaul traffic, considering the signal-to-noise ratio (SNR) constraint of UEs [8,19]. Elhattab et al. [20] jointly optimized the clustering and power control for the CoMP-JT in the cooperative non-orthogonal multiple-access networks. The objective is to maximize the network sum rate. Shami et al. [21] proposed a user-centric clustering approach and a bandwidth allocation algorithm for the CoMP. However, these works did not consider caches in cells.

Recently, some studies investigating the JT clustering problem have considered caches in cells [22,23,24]. Chen et al. [22] formulated the CoMP-JT clustering problem with the objective of maximizing the network throughput and proposed a binary particle swarm optimization algorithm to maximize the throughput. Yu et al. [23] considered mmWave links between cells in the JT clustering problem and proposed a two-stage algorithm to determine the JT clusters and routing paths. Yu et al. [24] proposed a heuristic algorithm to determine a JT cluster for each UE to minimize the backhaul data traffic.

In this article, we investigate the JT grouping problem considering caches in cells. The objective is to minimize the backhaul bandwidth consumption under quality-of-service (QoS) requirements and radio resource constraints. The contributions of this article are summarized as follows.

- We propose a genetic algorithm using a solution of the heuristic algorithm proposed in [24] as an initial state to reduce the time required for convergence and improve the algorithm’s performance. The value of this work can reduce the backhaul traffic when using CoMP-JT in specifications [2] and industries [25].
- The simulation results show that, compared with the JT grouping algorithm that considers caches [24] and the JT grouping algorithm that does not consider caches [15], the proposed genetic algorithm significantly reduces backhaul data traffic.

The remainder of this article is organized as follows: Section 2 describes the system model and formulates the problem. Section 3 explains the proposed algorithm. Section 4 presents the simulation results. Finally, Section 5 concludes the article.

## 2. System Model and Problem Formulation

In Section 2.1, we describe our considered system model. In Section 2.2, we formulate our target problem and define the notations.

### 2.1. System Model

The 5G networks should provide high video resolutions for UEs. To ensure QoS, UEs with bad channel conditions at cell edges require more radio resource blocks (RBs) than those at cell centers. Notably, an RB is a basic allocable unit. To overcome this issue, CoMP-JT is used to improve the SNR for UEs at cell edges. In CoMP-JT, we can select some cells clustered together in a JT group to deliver a video file using the same RBs for a UE. If a JT group uses some RBs, then the same RBs cannot be used by other JT groups to avoid interference.

However, each cell in the JT group must have the same video file. We assumed that each cell stores some video files. Therefore, a cell can obtain a video file either from a service provider through the network backhaul or directly from storage without using the network backhaul. The latter can reduce the backhaul data traffic. However, if we always select the cells that store the requested data by UEs to form a JT group, then the radio resources of the cells are quickly exhausted; on the other hand, if we select more cells without storing the requested data in a JT group, then it becomes necessary to transmit more data through the network backhaul. Therefore, the JT grouping problem has a trade-off between the consumption of RBs and backhaul bandwidth.

In addition, when a JT group has more cells, the SNR of a UE can generally be improved so that the number of RBs required to meet QoS can be saved. Different cells grouped into a JT cluster consume a different number of RBs to fulfill QoS. In the target problem, we must determine which and how many cells should join a JT group for serving a UE.

### 2.2. Problem Formulation

In this article, we investigate the JT grouping problem for using CoMP-JT in cellular systems, considering caches at cells. This problem is to minimize the total backhaul traffic under the data-rate requirement and limited RB constraints.

There is a set of UEs (denoted as *U*) in a network, and each UE u∈U requests one video file $f_{u}\in F$, where *F* is the set of selectable files. $D_{f_{u}}$ is the data-rate requirement of video file $f_{u}$. The set of cells deployed to serve the UEs is represented as *C* with *R* number of RBs. Each UE *u* is covered by a set of cells, denoted by $C_{u}$. Each UE *u* is restricted to connect with the cells in set $C_{u}$. The set of video files stored by cell c∈C is $F_{c}$. To transmit the required video file for each UE, we must determine a JT group (denoted as $J_{u}$) for each UE. In other words, $J_{u}$ is the set of cells used to serve UE *u*, $J_{u}\subseteq C_{u}$. The cells in set $J_{u}$ jointly transmit a file to UE *u*. The received SNR of UE *u* from cell group $J_{u}$ is written as:

(1) $${\mathrm{SNR}}_{u}=\frac{\sum_{c\in J_{u}}p_{c,u}\times h_{c,u}}{\sigma }$$

where $h_{c,u}$ is the channel gain of UE *u* from cell *c*, $p_{c,u}$ is the transmission power of cell *c* to UE *u*, and σ is the noise spectral density. Notably, we consider equal power allocation which can be replaced by any power allocation algorithm. Some studies investigated adaptive feedback schemes in neural networks [26,27,28].

The data rate provided by an RB transmitted by JT group $J_{u}$ for UE *u* can be calculated using the Shannon capacity formula [29,30] as shown in Equation (2):

(2) $$S_{u}=W\times \log_{2}\left(1+{\mathrm{SNR}}_{u}\right),$$

where *W* is the RB bandwidth. The number of RBs required to satisfy UE *u* with its data-rate requirement (denoted as $R_{u}$) is given by

(3) $$R_{u}=\frac{D_{f_{u}}}{S_{u}}.$$

If a cell has cached files requested by the UEs, the cell can transmit the file without consuming the backhaul bandwidth. We consider that the files cached by each cell are given. The backhaul traffic of each cell *c* can be calculated by:

(4) $$T_{c,u}=X_{c,u}\times D_{f_{u}},$$

where $X_{c,u}$ is an indicator function equal to 1 when cell *c* transmits data without caching file $f_{u}$ to UE *u*. In other words, when $X_{c,u}=1$, cell *u* generates backhaul traffic $D_{f_{u}}$; otherwise, $X_{c,u}$ is 0.

In this article, we aim to minimize the total backhaul data traffic by finding a JT group $J_{u}$ to serve each UE *u*. The objective function is represented as follows:

(5) $$\begin{array}{cc} & \min_{J_{u}}\sum_{u\in U}\sum_{c\in J_{u}}T_{c,u} \end{array}$$

(6) $$\begin{array}{cc} \mathrm{subject to}: & S_{u}\times R_{u}\geq D_{f_{u}},\forall u,\mathrm{and} \end{array}$$

(7) $$\begin{array}{cc} & \sum_{u\in U}I\left(c,I_{u}\right)\times R_{u}\leq R,\forall c\in C \end{array}$$

where

$$\begin{array}{c} I\left(c,I_{u}\right)\leq \left\{\begin{array}{cc} 1, & \mathrm{if}c\in I_{u}\cup J_{u}\\ 0, & \mathrm{otherwise} \end{array}\right. \end{array}$$

In the above formula, constraint (6) ensures that the data requirement of each UE *u* can be satisfied, and constraint (7) ensures that the RBs of each cell *c* assigned for UEs and interfered by other JT groups cannot surpass the total available RBs. $I_{u}$ is the set of cells that will be interfered by JT group $J_{u}$ transmitting data to UE *u*. The variables used in this problem are summarized in Nomenclature.

## 3. Methodology

In Section 3.1, we introduce the settings of the proposed genetic algorithm. In Section 3.2, we describe the proposed genetic approach to determine a JT group for each UE.

### 3.1. Settings of the Proposed Genetic Algorithm

A genetic algorithm is a method inspired by the concept of survival of the fittest in the biological evolution process [31]. The algorithm mimics natural selections by selecting better individuals for an environment and creating good offspring. By repeating the reproduction and natural selection processes, these individuals continuously evolve, and nearly optimal solutions may be obtained.

**Figure 1:**
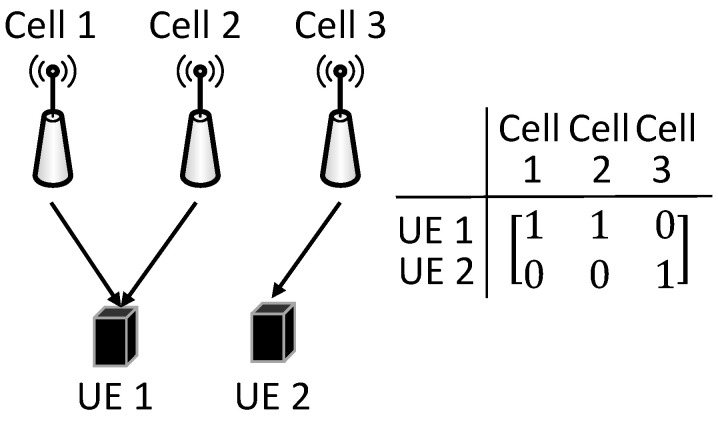
Example of chromosome representation for JT grouping.

A typical genetic algorithm includes population initialization, evaluation, selection, crossover, and mutation [31]. The population initialization function is to create initial solutions, called individuals, and each individual is encoded as a chromosome to represent the solution. Figure 1 shows example of a two-dimensional chromosome matrix used to represent a solution of the JT grouping problem. In this example, the JT group of UE 1 consists of cells 1 and 2, and the JT group of UE 2 consists of cell 3.

The evaluation function evaluates an individual’s fitness, which is calculated according to the objective function that measures the quality of a solution. After the evaluation, the individuals are appropriately selected as parents used for the following crossover and mutation. The selection operation stochastically selects individuals by the roulette-wheel selection based on fitness [32]. The individuals with higher fitness values are more likely to be chosen.

**Figure 2:**
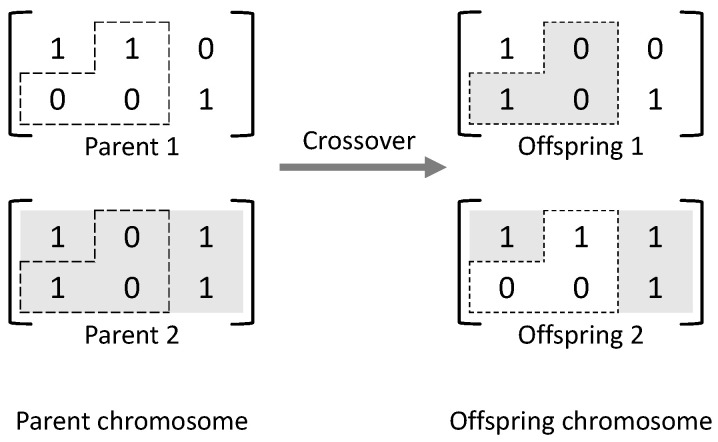
Example of crossover.

In the crossover operation, two parents exchange randomly chosen subsequences of their chromosomes to create a new pair of offspring. Multiple pairs of parents are selected to create multiple pairs of offspring chromosomes. Figure 2 is an example of crossover, where the area enclosed by the dashed line is a randomly selected subsequence. The two offspring chromosomes are a mixture of the parent chromosomes. The crossover operation is used to exploit better solutions to improve the performance of the algorithm.

A genetic algorithm that uses only the crossover mechanism can generate local optimum solutions. Mutation is an exploratory mechanism that helps discover global optimal solutions in genetic algorithms. The mutation operation randomly alters the partial gene values of a chromosome to randomly search different areas. Finally, a terminate function determines when a genetic algorithm stops: if the predefined termination condition is satisfied, the genetic algorithm stops; otherwise, the next generation repeats the above operations of evaluation, selection, crossover, and mutation.

### 3.2. Proposed Genetic Algorithm

In this section, we propose a genetic algorithm for our JT grouping problem. The concept of the proposed algorithm is described as follows. The proposed genetic algorithm uses the solution of the cache-enabled CoMP (*CEC*) algorithm proposed by Yu et al. [24] as the initial solution to reduce the algorithm’s execution time for convergence. Based on the initial solution, the designed Initial-Population() function attempts different probabilities to randomly interrupt the connection between a base station and a UE to generate multiple solutions. Then, the proposed genetic algorithm applies the crossover and mutation operations to generate solutions. After multiple solutions are created, we design the Evaluation() function to correct infeasible to feasible solutions and evaluate the backhaul traffic of each solution. An infeasible solution means that constraint (6) or (7) is not satisfied. After predefined generations are achieved, the algorithm selects the best solution with the minimum backhaul data traffic. Our proposed algorithm adopts the snapshot channel quality of each UE to determine a JT group for each UE. When the channel quality of a UE is severely changed, the proposed algorithm can be triggered to redetermine a JT group for the UE.

The pseudo-code for the proposed genetic algorithm is presented in Algorithm 1. In Line 1, we call the Initial-Population() function to generate our initial individuals (i.e., solutions). The inputs of the function are $J_{u}^{CEC}$ and *K*. $J_{u}^{CEC}$ is the solution of the *CEC* algorithm, and *K* is the predefined individual number (population size). Thus, we will generate *K* solutions. In Line 2, variable *g* is the generation index of the genetic algorithm. In Lines 3–16, we run *G* generations for our genetic algorithm. Determining the number of *G* generations involves a trade-off between the performance and execution time of the algorithm. In each generation (i.e., g=g+1 in Line 4), we call the Evaluation() function to correct infeasible to feasible solutions and evaluate the backhaul traffic of each solution in population set *P*. Variable *B* represents the set of backhaul traffic values corresponding to the set of solutions in the population set *P*. Variable *N* is the set of children and initialized as an empty set in each generation (Line 6).

**Algorithm 1:** Genetic Algorithm for JT Grouping.

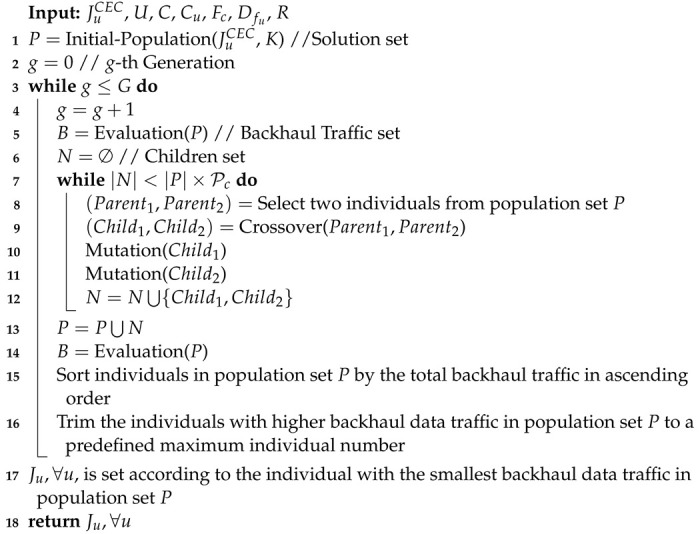



In Lines 7–12, we create $\left|P\right|\times P_{c}$ children using the crossover operation, where $P_{c}$ is the crossover probability to control the number of generated children. In Line 8, we select two solutions as two parents using the roulette-wheel selection policy. The solutions with lower backhaul traffic values are selected with higher probabilities. In Line 9, the Crossover() function is used to generate two children from the selected parents. In the Crossover() function, the two selected parents have the probability of $P_{c}$ to swap their subsequences of chromosomes by the one-point crossover operation to create two children, as shown in Figure 2. If the two parents do not execute crossover, we directly copy the two chromosomes of the two parents as the chromosomes of the two children.

In Lines 10 and 11, we execute the Mutation() function for each child separately. In the Mutation() function, each bit in the child’s chromosome matrix has a probability $P_{m}$ of being changed. If a bit value is altered from 0 to 1, it means that the base station represented by the bit joins the JT group of the UE; on the contrary, if a bit value is changed from 1 to 0, the corresponding base station leaves the JT group of the UE. Because each UE *u* can only connect with cell $c\in C_{u}$, we only mutated these elements in the chromosome matrix. After executing the mutation operation, the children generated by the crossover and mutation operations are added to the children set *N* (Line 12). When the while loop is completed, the generated children are added to the population set (Line 13). Then, we use the Evaluation() function to revise infeasible solutions, caused by the crossover and mutation operations, to feasible solutions and evaluate the backhaul data traffic of each solution in the population set *P* (Line 14). In Line 15, we sort the solutions in the population set *P* by the total backhaul traffic in ascending order. We then trim the solutions with higher backhaul data traffic in the population set to maintain a population size of *K* (Line 16). After generations, we select and return the solution with the fewest backhaul data traffic in the population set (Lines 17–18).

The Initial-Population() function (Algorithm 2) takes $J_{u}^{CEC}$, *U*, *C*, and *K* as inputs. $J_{u}^{CEC}$ is a solution generated by the *CEC* algorithm [24] and *K* is the number of solutions. This function is used to create multiple solutions from initial solution $J_{u}^{CEC}$. We use different disconnection probabilities $P_{d}$ to disconnect a cell *c* from the JT group $J_{u}^{CEC}$ of UE *u*. Whenever a solution is generated, the disconnection probability $P_{d}$ is increased in steps of $\frac{100\%}{K}$. After generating *K* solutions, we evaluate the performance of each solution and find the best solution corresponding to the best probability $P_{d}^{*}$. Then, we adopt the best disconnection probability $P_{d}^{*}$ to regenerate *K* solutions.

**Algorithm 2:** Initial-Population() Function.

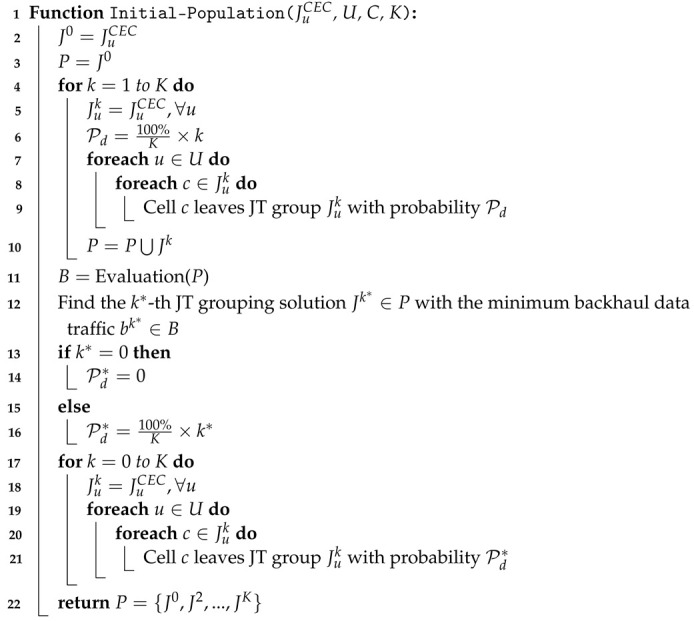



In Lines 2–3, we set our first solution $J^{0}$ as $J_{u}^{CEC}$, where $J^{k}$ is denoted as the *k*-th solution, and the first solution is added to the solution set *P*. In Lines 4–10, we generate another set of *K* solutions. We use $J_{u}^{CEC}$ as the initialization of the *k*-th solution $J_{u}^{k}$ (Line 5). We set the disconnection probability $P_{d}$ as $\frac{100\%}{K}\times k$ to generate the *k*-th solution. In Lines 7–9, for each UE *u*, each cell *c* leaves the JT group $J_{u}^{k}$ of UE *u* with probability $P_{d}$. In other words, when the value of *k* is larger, the disconnection probability is higher. Then, the generated *k*-th solution is added to the solution set *P* (Line 10).

After we create the *K* solutions, we call the Evaluation() function to evaluate the total backhaul traffic $b^{k}\in B$ of each solution $J^{k}\in P$ (Line 11), where *B* is the set of total backhaul traffic values corresponding to the solution set *P*. Then, we find the $k^{*}$-th solution with the minimum backhaul data traffic (Line 12). If $k^{*}=0$, the solution is the initial solution (i.e., $J_{u}^{CEC}$) so that the best disconnection probability $P_{d}^{*}=0$ (Lines 13–14); otherwise, the best disconnection probability $P_{d}^{*}$ is set to $\frac{100\%}{K}\times k^{*}$ (Lines 15–16). Next, we use the best disconnection probability to renew each solution in solution set *P* (Lines 17–21). Finally, this function returns *P* (Line 22).

The Evaluation() function (Algorithm 3) is used to adjust each infeasible solution to its corresponding feasible solution and to calculate the total backhaul consumption of each solution in *P*. The backhaul traffic set *B* is initialized as an empty set (Line 2). For the *k*-th solution, we check the JT group of each UE *u* (Lines 3–4). If the UE’s JT group is empty, its data requirement cannot be satisfied, and we firstly find a cell $c^{*}$ to cache the requested file $f_{u}$ with the highest SNR for UE *u*, where $c^{*}\in C_{u}$ and $f_{u}\in F_{c^{*}}$. We add this cell to the JT group of UE *u* (that is, $J_{u}^{k}=J_{u}^{k}⋃c^{*}$) in Lines 5–7. Then, if the number of $R_{u}$ RBs consumed by JT group $J_{u}^{k}$ for serving UE *u* is larger than the remaining $\beta_{c}$ RBs of a cell *c*, $\exists c\in I_{u}\cup J_{u}^{k}$, we should add more cells in the JT group to reduce $R_{u}$, where $\beta_{c}$ is the remaining RBs of cell *c* (Lines 8–9).

**Algorithm 3:** Evaluation() Function.

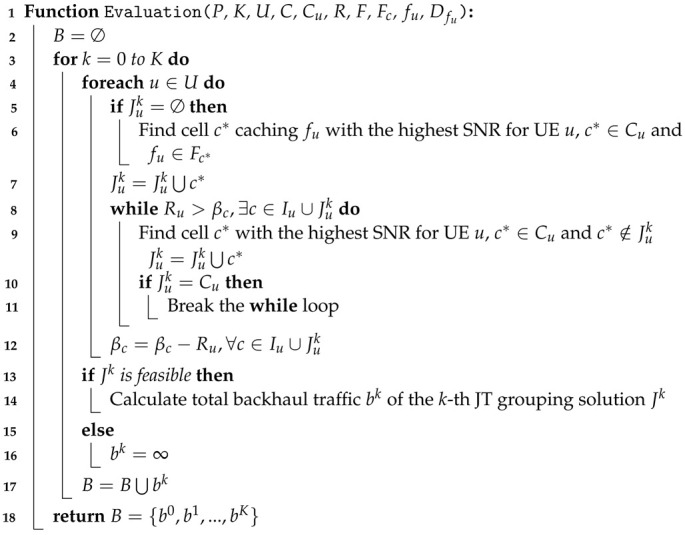



If all the cells covering UE *u* join to the JT group, it is impossible to reduce $R_{u}$ further, and we break the **while** loop (Lines 10–11). Each cell in the JT group and interfered by the JT group should reduce the consumed RBs ($R_{u}$) to serve UE *u* (Line 12). If the *k*-th solution is feasible after the adjustment, we calculate the total backhaul traffic $b^{k}$ of the *k*-th JT grouping solution; otherwise, $b^{k}$ is set to ∞ to indicate that it is not a feasible solution (Lines 13–16). Next, the traffic value $b^{k}$ of the *k*-th solution is added to backhaul traffic set *B* (Line 17). After each solution is evaluated, we return *B* (Line 18).

### 3.3. Property of the Proposed Algorithm

**Theorem** **1.** *The time complexity of Algorithm 1 is $O(|P||U|\hat{I}\gamma )$ for a generation, where $\hat{I}=\max_{\forall \;u,k}\left|I_{u}\cup J_{u}^{k}\right|$.*

**Proof.**  We analyze the time complexity of Algorithm 1 for a generation. In the Evaluation() function, we evaluate the backhaul traffic of |P| solutions. For a solution, there are |U| UEs. For a UE, if the RBs of a cell are insufficient, we find cells to join the JT group of the UE, where we should check at most $\hat{I}$ cells. Assume the checking time is γ; therefore, the Evaluation() function takes $O(|P||U|\hat{I}\gamma )$ time. Then, we should create |N| children. For generating two children, we call one Crossover() and two Mutation() functions. Let λ and ψ be the time complexity of the Crossover() and Mutation() function, respectively. Creating |N| children takes O(|N|(λ+2ψ)) time. The time complexity of Algorithm 1 is $O(|P||U|\hat{I}\gamma +|N|\left(\lambda +2\psi \right))$ for a generation. In addition, |P| is larger than |N|; |U| is a larger number than the other parameters, and the checking time is larger than the crossover and mutation functions. Thus, $O(|P||U|\hat{I}\gamma )$ dominates O(|N|(λ+2ψ)). Thus, the time complexity of Algorithm 1 is $O(|P||U|\hat{I}\gamma )$. □

## 4. Performance Evaluation

In Section 4.1, we introduce the compared baselines and the simulation setups. In Section 4.2, we explain our simulation results under different parameter settings.

### 4.1. Simulation Setups

In this section, we evaluate the performance of the proposed genetic algorithm to determine a JT group for each UE to minimize the total backhaul traffic. Our proposed genetic algorithm is namely the genetic JT grouping algorithm (GJGA). We compare the proposed genetic algorithm with the two baselines. The first baseline is the *CEC* algorithm proposed by Yu et al. [24]. The *CEC* algorithm is an iterative algorithm that gradually increases the size of each JT group. The size refers to the number of cells in a JT group. For a UE, the *CEC* algorithm firstly adds all the cells caching the requested file into the JT group because these cells do not consume the backhaul bandwidth. Then, if the RBs of some cells are insufficient, the algorithm adds more cells to the JT group until the number of cells is equal to the size. When the algorithm cannot find a feasible solution, the size is increased by one after each iteration. The second baseline is called backhaul traffic minimization (*BTM*), designed by Zhang et al. [15], without considering caches at the cells. The *BTM* algorithm selects cells with a higher SNR and adds them into each JT group until the data traffic requirement of each UE is satisfied. The *BTM* algorithm attempts to minimize the number of cells in each JT group to minimize the backhaul bandwidth consumption.

We developed our simulation via C programming language. Our simulation parameters are set in accordance with the study of [24]. We simulate a network environment with 132 base stations in a 2×0.85 km^2^ area. The diameter of each cell ranges from 400 to 800 m [33]. Under a 20 MHz bandwidth, the total number of RBs for each base station is set to 100. The cache size of each cell is set from 1000 to 4000. In other words, each cell can cache 1000 to 4000 files. The total number of selectable video files ranges from 8000 to 10,000. Each cell randomly stores the files in its storage, and each UE randomly selects one file. The data-rate requirement of a UE for watching a video is randomly selected from 599 to 735 kbps, measured from YouTube [24].

The number of the cell-edge UE is set from 100 to 600. Each UE is randomly located in a simulated environment. The SNR of each UE can be derived using Equation (1), where we consider that the path loss model is $35.2+35\log_{10}\left(\theta \right)$ in our channel model [34] and θ is in meters. Then, the data rate provided by an RB can be calculated according to Equation (2). Notably, we consider equal power allocation in this article. For our proposed genetic algorithm, we set the mutation and crossover probabilities to 0.05 and 50% (that is, $P_{m}=0.05\%$ and $P_{c}=50\%$), respectively. The number of solutions in our solution set is set as 30 (i.e., K=30).

### 4.2. Simulation Results

**Figure 3:**
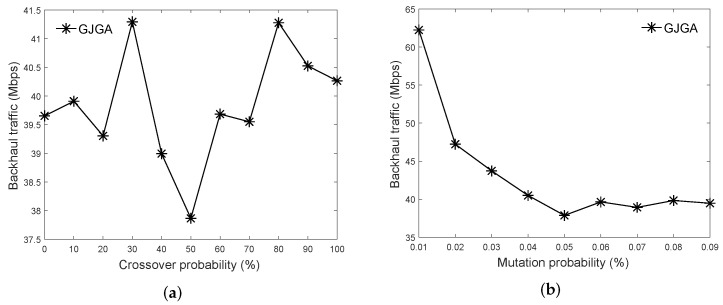
Effects of different crossover and mutation probabilities on the total backhaul traffic under 600 UEs, cache size of 4000, 8000 files, and 1000 generations. (**a**) Crossover under the mutation probability of 0.05%. (**b**) Mutation under the crossover probability of 50%.

**Figure 4:**
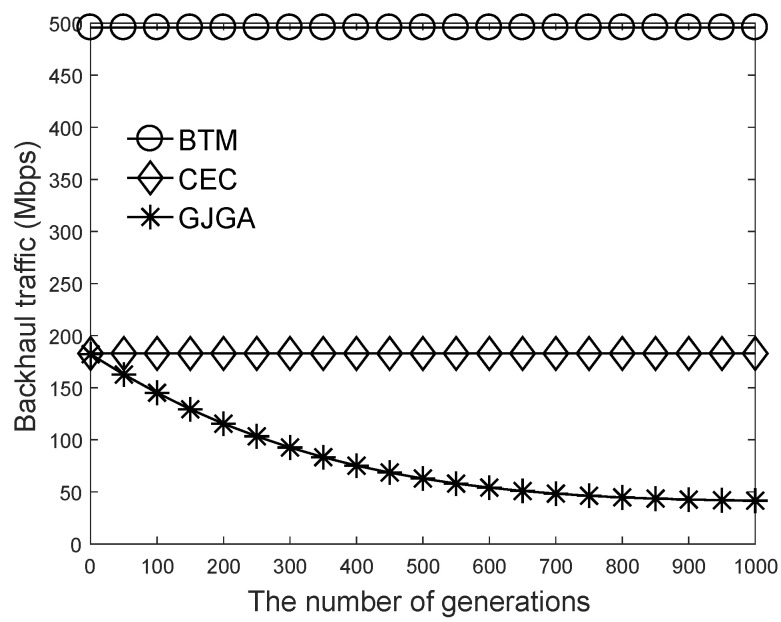
Effects of generations on the total backhaul traffic under 600 UEs, cache size of 4000, and 8000 files.

**Figure 5:**
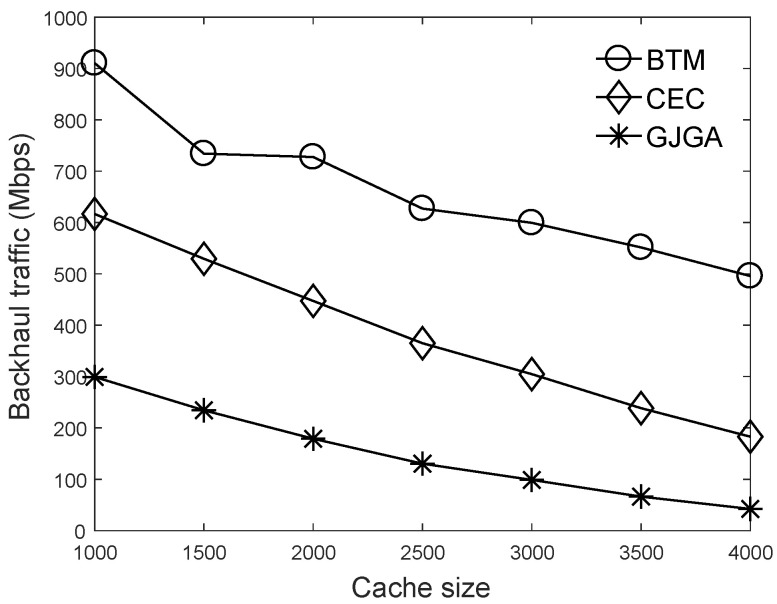
Effects of the cache size on the total backhaul traffic under 600 UEs and 8000 video files.

**Figure 6:**
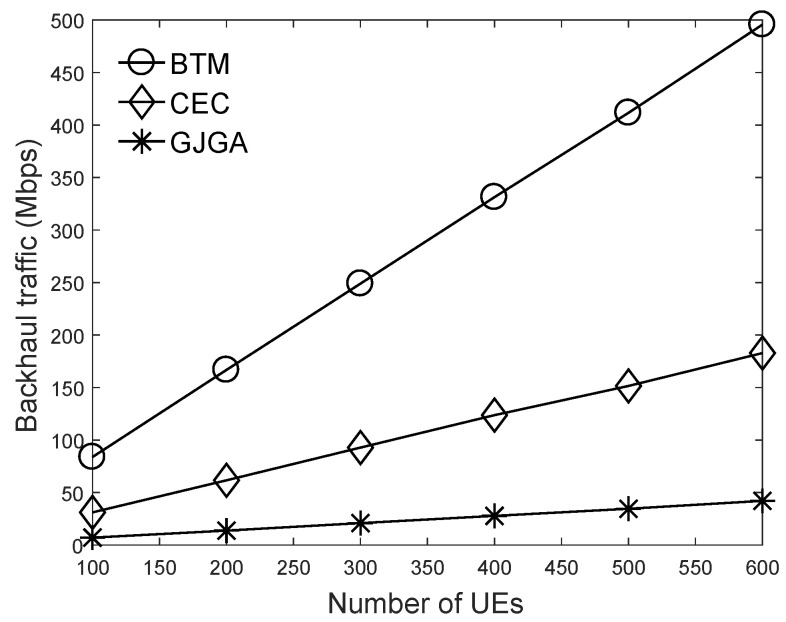
Effects of the UE number on the total backhaul traffic under the cache size of 4000 and 8000 video files.

**Figure 7:**
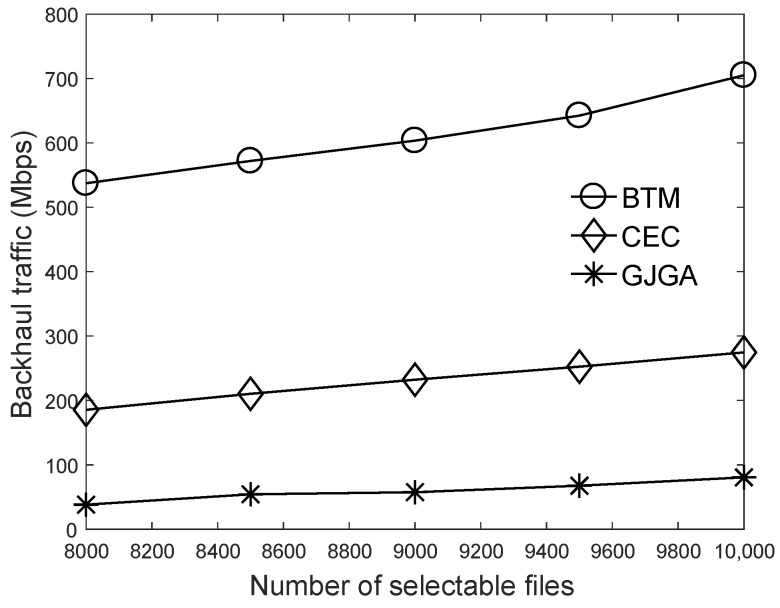
Effects of the number of selectable files on the total backhaul traffic under 600 UEs and the cache size of 4000.

We summarize the aim of the results in this section as follows. Based on the results of Figure 3, we set the crossover probability to 50% and the mutation probability to 0.05%. According to Figure 4, we suggest that our proposed genetic algorithm sets the number to 400 generations. Figure 5, Figure 6 and Figure 7 evaluate the cache size, the number of UEs, and the number of selectable files for the total backhaul traffic. The results show that our proposed algorithm can significantly reduce the backhaul traffic compared with the two baselines. The performance improvement is more evident when the selectable files are fewer or the cache size is larger.

Figure 3 shows the effects of the different crossover and mutation probabilities on the total backhaul traffic under the proposed genetic algorithm. As shown in Figure 3a, different crossover probabilities slightly affect the total backhaul traffic, approximately between 37.8 and 41.2 Mbps, when the number of generations is 1000. Based on our simulation results, we set the crossover probability to 50%. As shown in Figure 3b, when the mutation probability is 0.05%, the proposed algorithm exhibits the best performance. Therefore, we set the mutation probability to 0.05%.

Figure 4 evaluates the performance of the proposed algorithm for each generation. Because we adopt the solution of the *CEC* algorithm to generate our initial solutions, we can see that the *CEC* and *GJGA* algorithms have a similar performance in the first generation. As the number of generations increases, the total backhaul traffic decreases under the *GJGA* because our proposed algorithm finds better solutions in each iteration. When the number of generations is higher than 265, the backhaul traffic is lower than 100 Mbps, and the *GJGA* can reduce the backhaul traffic about 45.3% compared with the *CEC*. After 400 generations, the performance improvement ratio of our proposed genetic algorithm is lower than 0.1% at every generation. There is a trade-off between the execution time and the algorithm’s performance.

Figure 5 shows the effects of the cache size on the total backhaul traffic. When the cache size increases, the total backhaul traffic under the three algorithms is reduced because more files can be stored in the cells and be transmitted by cells without consuming the backhaul bandwidth. Our proposed *GJGA* algorithm can significantly reduce the total backhaul traffic consumed by the cells because it iteratively finds a suitable JT group for each UE to use the files caching in each cell efficiently. Therefore, when the cache size is larger, the performance improvement of the proposed algorithm is more evident. Although the *BTM* algorithm does not consider caches at cells, the backhaul traffic can still be decreased. This is because a requested file is more likely to be hit in the caches when the cache size is larger. Our proposed algorithm can reduce the backhaul traffic by up to 91 and 77% compared to the *BTM* and *CEC*, respectively.

Figure 6 investigates the number of UEs in the total backhaul traffic. As the number of UEs increases, the total backhaul traffic increases under the three algorithms. This is because more JT groups should serve more UE requests to generate more backhaul data traffic. Compared with the two baselines, the proposed algorithm significantly reduces the backhaul traffic to alleviate the burden of the network backhaul. The proposed genetic algorithm relies on our designed crossover, mutation, and evaluation functions to create multiple solutions and maintain better solutions for each generation. Therefore, the *GJGA* can iteratively adjust a suitable JT group for each UE to minimize the backhaul bandwidth consumption. Compared with the *CEC* algorithm considering caches, the proposed *GJGA* algorithm can reduce backhaul traffic by up to 71%. This result justifies our motivation that the JT grouping problem considering caches is vital for reducing the backhaul traffic.

Figure 7 shows the number of selectable files for the total backhaul traffic. When the number of selectable files increases, the total backhaul traffic increases under the three algorithms. When the cache size is given, more selectable files mean that lower ratios of files can be cached at each cell so that fewer files requested by UEs can be transmitted from the caches of the cells. The simulation result shows that the proposed algorithm still has the best performance with the minimum backhaul traffic because the *GJGA* can efficiently use the cells’ caches to reduce the backhaul burden under the limited RBs, even when some files may be rarely cached at a few cells. The proposed genetic algorithm considering caches finds a JT group for each UE to minimize the backhaul traffic. Our proposed algorithm can reduce the backhaul traffic by up to 92 and 79%, compared to the *BTM* and *CEC*, respectively. The performance improvement of the proposed genetic algorithm is more evident with fewer selectable files.

## 5. Conclusions

In cellular networks, the CoMP-JT is an important technology for improving network throughput. However, forming a JT group for a UE without considering caches at cells results in a network backhaul burden. This article investigates the JT grouping problem to minimize the backhaul traffic in cellular networks. We consider caches at cells in the JT group problem subject to the data requirement of each UE and the RBs of each cell. Then, we propose a genetic algorithm to solve this problem. To improve the execution time, the proposed genetic algorithm uses a solution of a heuristic algorithm (*CEC*) as our initial solution. The simulation results show that, compared with the two baselines, the proposed genetic algorithm obviously decreases the backhaul traffic and justifies that considering caches is vital for decreasing the backhaul traffic in the JT grouping problem. The limitation of this work is that we only consider single-layered video technologies. In future works, we will consider layered video technologies for the JT grouping problem.

## Data Availability

The data used to support the findings of this article are available upon request.

## Nomenclature

**Symbol**	**Depiction**
*U*	Set of UEs
*C*	Set of cells
$C_{u}$	Set of cells that cover UE *u*
$F_{c}$	Set of video files cached by cell *c*
*F*	Set of all selectable video files
$f_{u}$	Video file requested by UE *u*
$D_{f_{u}}$	Data rate of the video file $f_{u}$ requested by UE *u*
*R*	Total RBs of each cell
$J_{u}$	Set of cells serves UE *u*
$I_{u}$	Set of cells that will be interfered when JT group $J_{u}$ serves UE *u*
$R_{u}$	Number of RBs consumed by JT group $J_{u}$ for serving UE *u*
$S_{u}$	Data rate provided by an RB when JT group $J_{u}$ jointly transmits data to UE *u*
$T_{c,u}$	Backhaul traffic consumed by cell *c* for serving UE *u*
$X_{c,u}$	An indicator function which is 1 if cell *c* consumes backhaul bandwidth for serving UE *u*;
	otherwise, it is 0.

## Abbreviations

The following abbreviations are used in this manuscript:

UE	User equipment
SNR	Signal-to-noise ratio
CoMP	Coordinated multipoint
JT	Joint transmission
CS/CB	Coordinated scheduling/beamforming
QoS	Quality of service
RB	Resource block
